# Risk factors for multidrug resistance in tuberculosis patients with diabetes mellitus

**DOI:** 10.1186/s12879-022-07831-3

**Published:** 2022-11-11

**Authors:** Shuangping Li, Yalin Liang, Xinjun Hu

**Affiliations:** Infectious Diseases Department, The First Affiliated Hospital of Henan University of Science and Technology, Luoyang, Henan China

**Keywords:** Tuberculosis, Diabetes, Multidrug resistant, Risk factors, Prediction model

## Abstract

**Objective:**

To study the risk factors and prediction models of multidrug resistance in patients with tuberculosis and diabetes and those with a history of tuberculosis treatment.

**Methods:**

A total of 256 tuberculosis patients with diabetes who were registered in Luoyang city, Henan Province, from January 2018 to December 2021. Logistic regression analysis was performed to analyse the risk factors for multidrug resistance. ROC curves were used to analyse the predictive model for multidrug resistance.

**Results:**

Age < 65 years old, HbA1c, and a history of tuberculosis treatment were independent risk factors for multidrug resistance in patients with tuberculosis and diabetes (P < 0.05). The area under the ROC curve of predictive model for MDR was 0.878 (95% CI (0.824, 0.932)). Age < 65 years old and HbA1c were independent risk factors for MDR in patients with TB and diabetes with a history of TB treatment. The area under the ROC curve of predictive model for MDR was 0.920 [95% CI (0.831, 0.999)].

**Conclusion:**

The predictive model had certain prediction value for the risk of multidrug resistance in patients with tuberculosis and diabetes.

## Background

Tuberculosis (TB) is one of the deadliest infectious diseases in the world. Despite a slow decline in its incidence, tuberculosis remains a major global public health problem. The growing prevalence of diabetes mellitus (DM) has emerged as one of the major global health challenges and may lead to an increase in the burden of TB. China has a heavy burden of TB with DM (patients had both tuberculosis and diabetes) [1]. Studies have shown that due to the impaired immune function of DM patients, the risk of active TB increases by 3 times [2]. Approximately 15% of TB cases worldwide are attributed to DM [3], and approximately 17% of TB cases in China are attributed to DM [4]. DM also leads to the occurrence of multidrug-resistant tuberculosis (MDR TB) to a certain extent [5], which is positively correlated with the occurrence of MDR TB [6]. MDR TB is defined as tuberculosis that is resistant to at least both rifampicin and isoniazid [7]. The diagnosis and treatment of MDR TB is an ongoing global public health challenge [8]. Early identification of risk factors for MDR TB in patients with TB and DM could reduce the risk of MDR TB and can simultaneously facilitate early diagnosis and treatment of patients. Studies have reported that in global the incidence rates of multidrug resistance in newly diagnosed and previously treated TB patients are 3.6% and 17%, respectively [9]. In China, the incidence rates of multidrug resistance in newly diagnosed and previously treated TB patients are 7.1% and 24% [10]. A history of TB treatment is an independent risk factor for the occurrence of multidrug resistance, therefore, patients with a history of TB treatment warrant greater attention. The main purpose of this study was to study the risk factors and predictive models of multidrug resistance in patients with TB and DM and those with a history of TB treatment.

## Methods

### Patient selection

The study was carried out as a nationwide retrospective register-based cohort study including all adult patients (> 18 years) notifed with TB and DM (including T1DM and T2DM) in Luoyang city, Henan Province, from January 2018 to December 2021. Both patients with pulmonary TB (PTB) and extrapulmonary TB (ETB) were included in the study. Inclusion criteria: Patients diagnosed with TB and DM. Inclusion criteria of TB dependedon the Chinese guidelines for the diagnosis and treatment of TB (2020 version) [11]: (1) The sputum smear was positive for acid-fast bacilli; (2) Mycobacterium tuberculosis cogroup was cultured in sputum, bronchoalveolar lavage fluid or pleural effusion; (3) The nucleic acid of Mtb and/or Mtb culture was positive in sputum, bronchoalveolar lavage fluid, or pleural effusion; (4) the pathological staining of lung tissue specimens was positive for acid-fast bacilli, or the nucleic acid of Mtb was positive in lung tissue specimens from the lesion site. The diagnosis of pulmonary TB could be clarified if any of the above four items were met. The patient was diagnosed with MDR TB by sputum culture and DST, GeneXpert MTB/RIF1, or only sputum culture and DST demonstrated at least simultaneous resistance to isoniazid and rifampicin. Diagnostic criteria for DM: (1) FPG ≥ 7.0 mmol/L (126 mg/dL); (2) A 2-h plasma glucose level of 200 mg/dL (11.1 mmol/L) or higher during OGTT (the 75-g oral glucose tolerance test) or HbA1c ≥ 6.5% [12, 13]. Exclusion criteria: Patients without sputum culture and drug sensitivity test; patients with an unclear diagnosis of DM, and patients with incomplete data.

### Data collection

The following clinical data of patients were collected: sex, age, body mass index (BMI), smoking history, history of alcoholism, history of TB treatment (including a end of the original treatment course, treatment course interruption, treatment failure, using a non-standard TB regimen), DM duration, co-morbidities (chronic obstructive pulmonary disease, coronary heart disease, hypertension, cerebral infarction, HIV), treatment options for diabetes, tuberculous pleuritis, Chest CT showed cavity, laboratory indices (HbA1c, hypersensitive c-reactive protein, erythrocyte sedimentation rate, white blood cell count, neutrophils, haemoglobin, platelets, glutamic-pyruvic transaminase (ALT), glutamic oxaloacetic transaminase(AST), albumin, uric acid, creatinine). The above data are the data of patients at the time of registration.

### Statistical analysis

SPSS 23.0 and MedCalc were used for statistical analysis. Means were compared using an independent t test. Count data were expressed as percentages, and differences in these characteristics between cases and controls were analysed using the chi-square test. Risk factors were analysed by multivariable logistic regression analysis. Receiver operating characteristic (ROC) curves and the area under the curve (AUC) were used to evaluate the predictive value of each risk factor and its combination in the occurrence of multidrug resistance in TB with DM. A p-value < 0.05 was considered statistically significant.

## Results

**Fig. 1:**
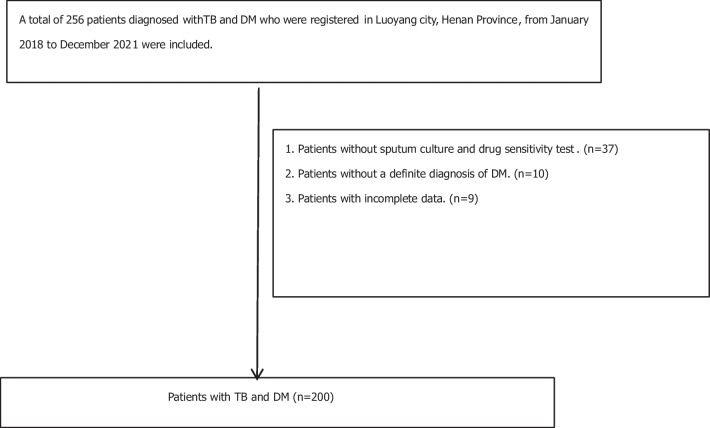
Flow chart of the patients enrolled in the study

A total of 256 patients were registered with TB with DM. According to the exclusion criteria, 200 patients with TB and DM were included, including 156 non-MDR TB patients and 44 MDR TB patients. Among the 200 patients with TB and DM, 44 patients had a history of TB treatment, including 18 patients with non-MDR TB and DM and 26 patients with MDR TB and DM. 44 patients of had a treatment history of TB including 23 cases had a end of the original treatment course, 8 cases had a treatment course interruption, 10 cases had a treatment failure, 3 cases had a non-standard TB regimen. The inclusion flow chart is shown in Fig. 1.

### Comparison of general data between patients with non-MDR TB and DM and patients with MDR TB and DM

Previous studies showed that MDR-TB patients were more likely to be younger than 65 years old [14–17], so we put the age cut-off at 65 years old. As shown in Table 1, the percentage of patients with MDR TB and DM was significantly higher than the non-MDR TB and DM in aged < 65 years old (p < 0.05), the percentage of MDR TB and DM patients with a smoking history and TB treatment history were significantly higher than non-MDR TB and DM (p < 0.05), and the level of HbA1c of MDR TB and DM was significantly higher than non-MDR TB and DM (p < 0.05).

**Table 1:** The demographic and clinical parameters of patients with TB and DM

	Non-MDR TB with DM (n = 156)	MDR TB with DM (n = 44)	P value
Male, n (%)	127 (0.81)	35 (0.79)	0.781
Age < 65 years old, n (%)	88 (0.56)	36 (0.81)	0.002
Body mass index (BMI)	21.33 ± 3.60	21.11 ± 3.66	0.720
Smoking history, n (%)	55 (0.35)	23 (0.52)	0.041
History of alcoholism, n (%)	21 ( (0.13)	9 (0.20)	0.251
History of TB treatment, n (%)	18 (0.11)	26 (0.59)	0.001
DM duration (month)	44.87 ± 17.91	56.72 ± 16.85	0.544
Coronary heart disease, n (%)	14 (0.08)	5 (0.11)	0.633
Chronic obstructive pulmonary disease, n (%)	16 (0.10)	3 (0.06)	0.492
Hypertension, n (%)	38 (0.24)	8 (0.18)	0.390
Cerebral infarction, n (%)	20 (0.13)	7 (0.16)	0.596
HIV,n (%)	7 (0.04)	3 (0.07)	0.531
Treatment of diabetes (insulin), n (%)	43 (0.27)	10 (0.23)	0.521
Empty hole, n (%)	124 (0.79)	39 (0.88)	0.167
With pleural effusion, n (%)	39 (0.25)	12 (0.27)	0.760
HbA1c, %	8.94 ± 2.45	11.12 ± 2.46	0.001
Erythrocyte sedimentation rate, mm/h	41.87 ± 26.03	43.62 ± 20.58	0.725
Hypersensitive C-reactive protein, ng/L	47.61 ± 37.28	36.14 ± 27.15	0.470
White blood cell count, 10^9^	7.33 ± 2.91	7.01 ± 2.39	0.508
Neutrophil count, 10^9^	5.37 ± 2.72	4.98 ± 2.21	0.377
Lymphocyte count	1.21 ± 0.566	1.28 ± 0.54	0.445
Haemoglobin, g/L	117.01 ± 20.57	120.79 ± 18.84	0.273
Platelet, 10^9^	302.32 ± 120.54	299.16 ± 102.01	0.874
ALT, U/L	21.69 ± 19.61	20.25 ± 12.50	0.678
AST, U/L	25.93 ± 13.94	26.38 ± 17.23	0.920
Albumin, g/L	32.90 ± 6.18	33.88 ± 6.05	0.350
Uric acid, umol/L	277.91 ± 127.43	324.55 ± 144.27	0.059
Creatinine, umol/L	64.23 ± 51.17	60.72 ± 26.04	0.662

*HIV* human immunodeficiency virus, *ALT* glutamic-pyruvic transaminase, *AST* glutamic oxaloacetic transaminase, *DM* diabetes mellitus, *TB* tuberculosis, *MDR* multi-drug resistant

### Risk factors for multidrug resistance in TB and DM

Age < 65 years old, smoking history, a history of TB treatment, HbA1c and sex were entered into a multivariable logistic regression analysis. Age < 65 years old, HbA1c level and a history of TB treatment were independent risk factors for multidrug resistance in patients with TB and DM (p < 0.05), as shown in Table 2.

**Table 2:** Multivariable logistic regression analysis of risk factors for multidrug resistance in patients with TB and DM

	B	SE	p value	Exp (B)	95%CI
Male	0.970	0.626	0.121	0.379	0.111, 1.293
Age < 65 years old	− 1.322	0.588	0.025	0.267	0.084, 0.844
HbA1c	0.434	0.109	0.000	1.543	1.245, 1.911
Smoking history	− 0.836	0.514	0.104	0.433	0.158, 1.187
History of TB treatment	− 3.371	0.559	0.000	0.034	0.011, 0.103

B, regression coefficient; SE, standard error

### Predictive value of age < 65 years old, HbA1c, a history of TB treatment, and the three risk factors combined for multidrug resistance in patients with TB and DM

**Fig. 2:**
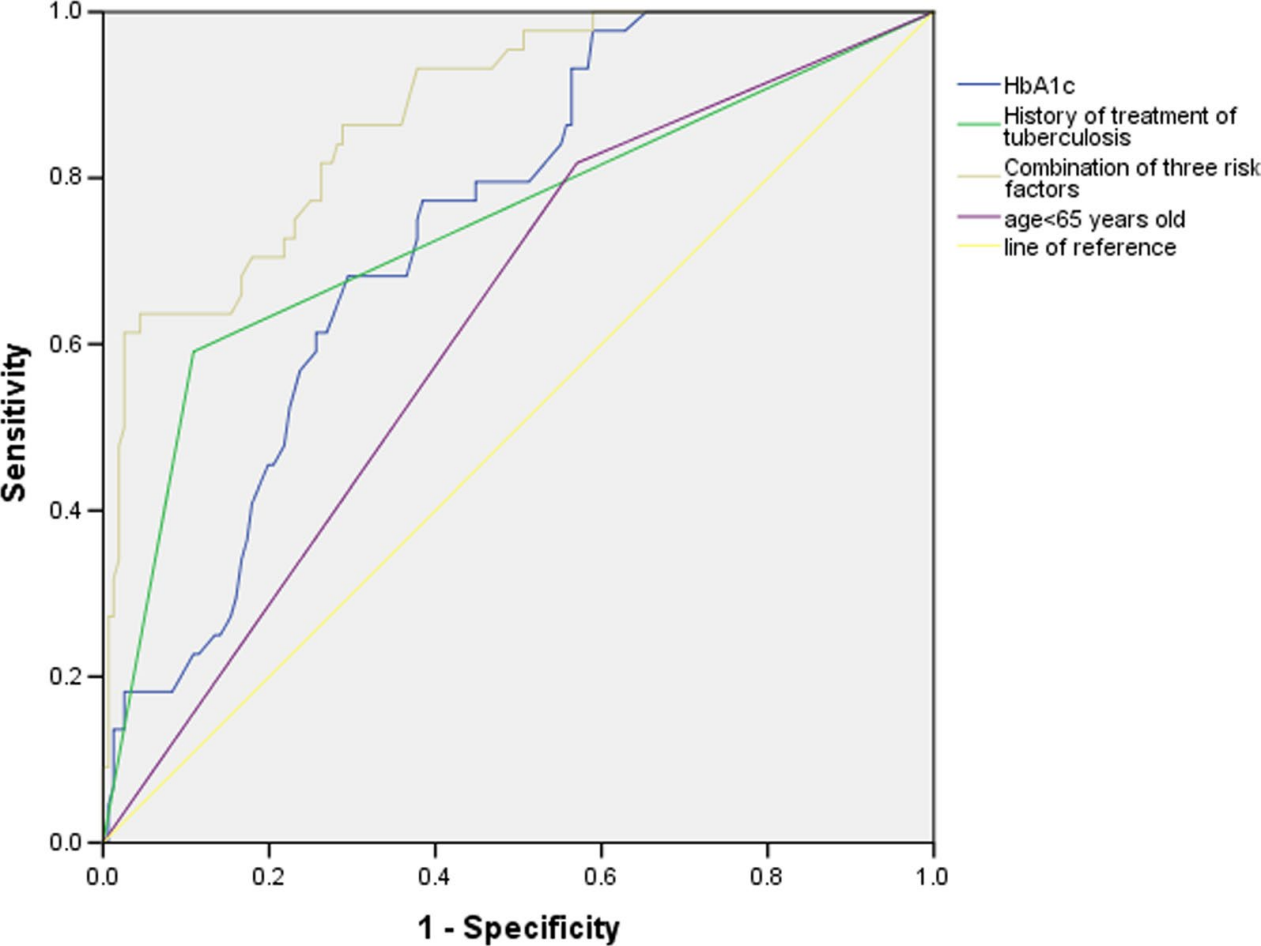
ROC curves for age < 65 years old, Hb1Ac, a history of TB treatment, and all three risk factors combined for multidrug resistance in patients with TB and DM

ROC curves for the predictive value of age < 65 years old, HbA1c, a history of tuberculosis treatment and the three risk factors combined for multidrug resistance in TB and DM were drawn (Fig. 2). The AUC of age < 65 years old was 0.624 (95% CI (0.536, 0.712)), the sensitivity was 81.82%, and the specificity was 42.95%. The AUC of HbA1c was 0.739 (95% CI (0.666, 0.812)), and the sensitivity and specificity were 77.27% and 61.54%, respectively, when the optimal cut-off value was 9.3%. The AUC of TB treatment history was 0.741 (95% CI (0.648, 0.834)), the sensitivity was 59.09%, and the specificity was 89.1%. The AUC of the combination of age < 65 years old, HbA1c and a tuberculosis treatment history was 0.878 (95% CI (0.824, 0.932)), the sensitivity was 63.64%, and the specificity was 95.91%.

### Comparison of general data between patients with non-MDR TB and DM and patients with MDR TB and DM with a history of TB treatment

As shown in Table 3, the percentage of MDR TB and DM was significantly higher than non-MDR TB in aged < 65 years old (p < 0.05), and HbA1c of MDR TB patients was significantly higher than non-MDR TB (p < 0.05).

**Table 3:** Comparison of general data between patients with non-MDR TB and DM and patients with MDR TB and DM with a history of TB treatment

	Non-MDR TB and DM with a history of TB treatment (n = 18)	MDR TB and DM with a history of TB treatment (n = 26)	P value
Male, n (%)	16 (0.88)	21 (0.81)	0.469
Age < 65 years, n (%)	5 (0.28)	21 (0.81)	0.000
Body mass index (BMI)	20.48 ± 3.67	20.81 ± 3.75	0.777
Smoking history, n (%)	4 (0.22)	13 (0.50)	0.063
Treatment of diabetes (insulin), n (%)	7 (0.38)	7 (0.27)	0.318
HbA1c, %	8.17 ± 1.32	10.72 ± 2.64	0.001

DM, diabetes mellitus; TB, tuberculosis; MDR, multi-drug resistant

### Risk factors for multidrug resistance in patients with TB and DM with a history of TB treatment

Age < 65 years old, HbA1c and sex were entered into a multivariable logistic regression analysis. Age < 65 years old and HbA1c were independent risk factors for multidrug resistance in patients with TB and DM with a history of TB treatment (p < 0.05), as shown in Table 4.

**Table 4:** Multivariable logistic regression analysis of risk factors for multidrug resistance in patients with TB and DM with a history of TB treatment

Variables	B	SE	*p* value	Exp (B)	95%CI
Male	− 0.883	1.611	0.584	0.413	0.018, 9.723
Age < 65 years	− 3.132	1.123	0.005	0.044	0.005, 0.394
HbA1c	1.194	0.458	0.009	3.301	1.347, 8.093

B, regression coefficient; SE, standard error

### Predictive value of age < 65 years old, HbA1c, and the two risk factors combined for multidrug resistance in TB and DM

**Fig. 3:**
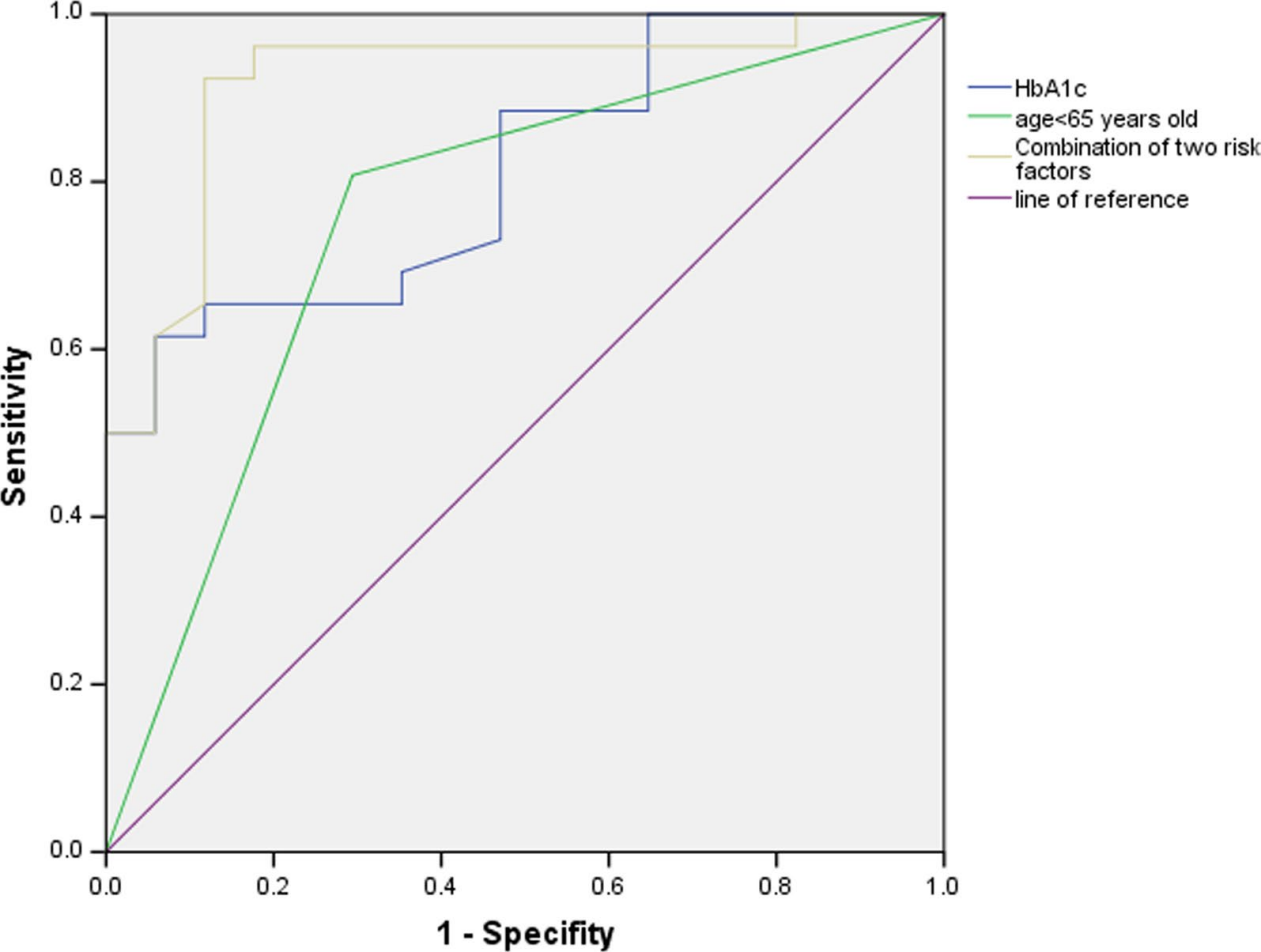
ROC curves for the predictive value of age < 65 years old, HbA1c, and the two risk factors combined for multidrug resistance in patients with TB and DM with a history of TB treatment

ROC curves of the predictive value of age < 65 years old, HbA1c, and the combination of the two risk factors for multidrug resistance in patients with TB and DM with a history of TB treatment were constructed (Fig. 3). The AUC of age < 65 years old was 0.757 (95% CI (0.601, 0.912)), the sensitivity was 80.77%, and the specificity was 70.59%. The AUC of HbA1c was 0.812 (95% CI (0.687, 0.938)), the sensitivity and specificity were 61.54% and 94.12%, respectively, when the optimal cut-off value was 9.9%. The AUC of age < 65 years old and HbA1c combined was 0.920 (95% CI (0.831, 0.999)), the sensitivity was 92.31%, and the specificity was 88.24%,

## Discussion

The global burden of combined TB-DM is high, with a global prevalence of 16% and rates of 24% in North America, 23% in Oceania, 17% in Asia, 11% in South America, 7% in Africa and 6% in Europe [3]. Diabetes may lead to immune dysfunction and changes in cytokine levels and the activation status of macrophages. Meanwhile, a high-glucose acidic environment may be conducive to the growth of MDR TB, which may reduce the efficacy of anti-tuberculosis drugs and increase the risk of drug resistance [18–20]. MDR TB is difficult to treat and has a high mortality rate. Some studies have shown that 20% of MDR TB patients died during treatment [21]. Previous studies had focused more on risk factors for MDR-TB. However, few studies had been conducted on the risk factors for multidrug resistance in TB and DM. Early identification of risk factors for MDR TB in patients with TB and DM could reduce the risk of MDR TB and can simultaneously facilitate early diagnosis and treatment of patients.

Former studies had shown that alcoholism, smoking, irregular treatment and lung cavities, age under 65 years, diabetes and HIV positivity may be risk factors for MDR-TB [10, 22]. Bocar Baya [24] concluded age < 65 years, previous TB treatment failures, number of previous TB treatment courses < 2, close contact with TB patients were risk factors for MDR-TB. However, few studies have been conducted on the risk factors for multidrug resistance in TB and DM. In our study, the HbA1c level was an independent risk factor for multidrug resistance in TB and DM. In our study, the prediction value of multidrug resistance was higher when HbA1c was 9.3%. Mengyuan Lyu [24] have concluded that HbA1c grade of 7% has higher predictive value for multidrug resistance. The reasons are as follows: a high HbA1c level is considered a factor leading to tissue hypoxia by increasing the affinity of haemoglobin for oxygen, while a low tissue oxygen concentration can lead to oxidative stress. An increased HbA1c may lead to resistance to isoniazid by contributing to oxidative stress [25]. Hyperglycaemia can reduce the concentration of anti-tuberculosis drugs in the blood by delaying drug absorption and promoting drug clearance, and the reduction in the drug concentration is conducive to the development of drug resistance to a large extent [20]. There were meta-analyses showed that MDR-TB patients were more likely to be younger than 65 years old [17]. A study had shown that aged between 45 and 64 years was risk factors for acquired drug resistance among retreated TB-DM cases, 65 ≥ years old was protective factors for acquisition of drug resistance [14]. Our study concluded that age < 65 years old was an independent risk factor for multidrug resistance in patients with TB and DM, which was consistent with FAUSTINI A et al. [17].The considered reason was that MDR-TB patients were younger than non-MDR-TB patients [23]. Previous studies had found that MDR-TB was more common in patients younger than 65 years of age given that they were busier with work or study than older patients and subsequently more exposed to the risk of MDR-TB [15, 16].^.^

There were two mechanisms for DR-TB: (i) acquired (or secondary) resistance: *Mycobacterium tuberculosis* strains develop resistance during TB treatment, and acquired resistance was defined as occurring when baseline DST result showed susceptibility in vitro, the final DST result showed resistance in vitro. (ii) transmitted (or primary) resistance: infection with a drug-resistant strain [14] .It had been widely accepted that had an inadequate treatment history were the major reasons of acquired drug resistance in TB patients [14]. Among the 44 MDR TB with DM patients in our study, 26 had a history of TB treatment, 26 were considered as acquired MDR and 18 were primary MDR. Prior treatment had been one of the most consistent independent risk factors for MDR-TB [23]. Our study found that a history of TB treatment was an independent risk factor for drug resistance in patients with TB and DM. As TB patients with a history of TB treatment had a significantly increased probability of developing multidrug resistance, some studies in China had shown that the incidence of MDR-TB in re-treated tuberculosis patients was 26.3% (23.1%–29.7%) [14]. However, few previous study had focused on the risk factors for MDR-TB in patients with TB and DM with a history of TB treatment separately. In our study, risk factors for multidrug resistance in patients with TB and DM with a history of TB treatment were analyzed separately, age < 65 years old and HbA1c remained independent risk factors for multidrug resistance.

Mengyuan Lyu [24] concluded that the AUC of modle of HbA1c rating (7%), age, erythrocyte sedimentation rate, hemoglobin, and C-reactive protein predicting MDR TB was 0.754, had a better predictive value. In our study, combining age < 65 years old, HbA1c and a TB treatment history yielded a certain predictive value for multidrug resistance in patients with TB and DM, the AUC was 0.878. Combining age < 65 years old and HbA1c yielded higher predictive value for multidrug resistance in patients with TB and DM with a history of TB treatment, the AUC was 0.920.

### Limitations

First, we separately analyzed the risk factors for MDR in patients with TB and DM who had a history of TB treatment, however due to the small number of patients with primary MDR, we did not analyze the risk factors for primary MDR separately. Second, although we had collected all TB-DM cases with susceptibility data and information in Luoyang city, Henan Province, from January 2018 to December 2021, the sample size was still limited. More appropriate cases need to be enrolled in order to gain a higher validity and reliability of our findings.

## Conclusion

Our study concluded that age < 65 years old, HbA1c and a history of TB treatment were independent risk factors for MDR in patients with TB and DM. The combination of the above three risk factors had certain predictive value for multidrug resistance in patients with TB and DM. Age < 65 years old and HbA1c were independent risk factors for multidrug resistance in patients with TB and DM who had a history of TB treatment. The combination of the above two risk factors had high predictive value for the risk of multidrug use in patients with TB and DM who had a history of TB treatment. The predictive model had certain prediction value for the risk of multidrug resistance in patients with tuberculosis and diabetes.

## Data Availability

All data generated or analyzed during this study are included in this published article.
